# Swedish consensus regarding difficult pre-hospital airway management: a Delphi study

**DOI:** 10.1186/s12873-024-01013-x

**Published:** 2024-05-27

**Authors:** Anton Modée Borgström, Denise Bäckström

**Affiliations:** 1 Department of Anaesthesiology and Intensive Care, Capio St. Göran’s Hospital, Stockholm, 112 19 Sweden; 2 Department of Biomedical and Clinical Sciences, Linköping University, Linköping, 581 83 Sweden; 3 Capio Akutläkarbilar, Stockholm, Sweden

**Keywords:** Prehospital, Airway management algorithm, Difficult airway, Sweden

## Abstract

**Background:**

The aim of this study was to establish a consensus among experts in prehospital work regarding the management of difficult airways in prehospital care in Sweden. The results were subsequently used to develop an algorithm for handling difficult airway in prehospital care, as there was none available in Sweden prior to this study.

**Methods:**

This two-round Delphi study was conducted by forming an expert panel comprising anesthesiologists and anesthesia nurses working in prehospital setting in Sweden. The expert panel responded digital forms with questions and statements related to airway management. The study continued until consensus was reached, defined as more than 70% agreement. The study took place from December 4, 2021, to May 15, 2022.

**Results:**

In the first round, 74 participants took part, while the second round involved 37 participants. Consensus was reached in 16 out of 17 statements. 92% of the participants agreed that an airway algorithm adapted for prehospital use is necessary.

**Conclusions:**

The capacity to adapt the approach to airway management based on specific pre-hospital circumstances is crucial. It holds significance to establish a uniform framework that is applicable across various airway management scenarios. Consequently, the airway management algorithm that has been devised should be regarded as a recommendation, allowing for flexibility rather than being interpreted as a rigid course of action.

This represents the inaugural nationwide algorithm for airway management designed exclusively for pre-hospital operations in Sweden. The algorithm is the result of a consensus reached by experts in pre-hospital care.

**Supplementary Information:**

The online version contains supplementary material available at 10.1186/s12873-024-01013-x.

## Background

One of the main contributors to preventing death and permanent injuries in critically ill and injured patients is the establishment of an appropriate airway and the prevention of hypoxia [1, 2]. Establishing a free airway can be difficult, and a commonly used definition of a difficult airway includes difficult mask ventilation, difficulty performing endotracheal intubation (ETI), or both [3]. Patient factors, the skills of the healthcare provider, and the clinical situation all influence the difficulty of establishing a free airway [3]. Airway management checklists, education, and better equipment have improved airway management-related mortality and morbidity in an in-hospital setting [4].

In Sweden, the pre-hospital emergency medical service (EMS) staff consists of nurses and/or nurse assistants [5]. Additionally, there are physician-staffed teams with specially trained physicians, usually experienced anesthesiologists, serving as a supplement [5]. These units operate either helicopters or rapid reaction cars. A review of pre-hospital emergency healthcare in Scandinavia reveals that the incidence of critical illness and injury requiring pre-hospital anesthesiologists is 25–30/10,000 persons/year [6]. An overview from 2010 indicated that the rate of advanced life support interventions in Sweden was 46 per 100,000 inhabitants per year [5]. According to an international multicenter observational study, 16% of primary missions conducted by physician-staffed helicopter teams required an advanced pre-hospital airway intervention, of which 92% necessitated ETI [7]. In Sweden intubation has been shown to be one of the most common procedures provided by prehospital physicians when supporting the ambulance service [8].

The Fourth National Audit Projects (NAP4) from 2011 analyzed 2.9 million general anesthetics to estimate the incidence of major complications of airway management in the UK [9]. NAP4 revealed that events related to anaesthetics resulted in a mortality rate of 5.6 per million general anesthesias [9]. However, this estimation assumes that all events were captured (statistical analysis suggests that only 25% of all events were reported) and that these finding are specific to an in-hospital setting with better monitoring, resources, equipment, and a safer environment compared to a pre-hospital setting [9].

The NAP4 recommends the use of an airway management algorithm [9], which could lead to improved patient outcomes and the prevention of airway-related complications [9]. There are numerous comprehensive and widely used algorithms for difficult airway management published by recognized airway societies [10]. A review of the existing algorithms for difficult airway management revealed an overwhelmingly similar structure but differences in terminology. These algorithms typically consist of a four-step flow chart, beginning with ETI and progressing to mask ventilation, supraglottic airway device (SAD), and, as a final option, rescue emergency surgical airway [10].

Combes et al. observed that in cases of an unanticipated difficult airway, where direct laryngoscopy was not feasible, the performer had to resort to an alternative approach [11]. They proposed a simple algorithm for managing difficult airways, suggesting the use of a gum elastic boogie as the primary method and an intubating laryngeal mask airway as the secondary option [11]. This approach resulted in a success rate of 98% for managing unanticipated difficult airways [11]. It is worth noting that complications related to ETI were high, with a rate of 52%, with esophageal intubation (36%) and arterial oxygen desaturation (26%) being the most common complications [11].

The Scandinavian Society of Anaesthesiology and Intensive Care Medicine (SSAI) conducted a literature review and based on that, developed practical guidelines for airway management in a pre-hospital setting in Scandinavia, according to provider training [12]. They suggest that all EMS should be proficient in basic airway maneuvers, while intermediately trained providers should use SAD, but emphasize that ETI should only be performed by advanced trained providers [12]. SAD may be utilized by advanced trained providers in cases where ETI is not feasible or in selected indications. Videolaryngoscopy is recommended when difficult direct laryngoscopy is anticipated or when direct laryngoscopy fails [12]. In situations where the patient cannot be intubated or ventilated, cricothyroidotomy should be performed by advanced trained providers [12].

The association of Anaesthetists of Great Britain and Ireland has also issued guidelines for safe pre-hospital anesthesia [13]. They emphasize the importance of having a written plan and practicing it for airway management and failed ETI, which should be standardized and easily reproducible by all pre-hospital organizations [13]. Furthermore, they stress the significance of maintaining the same standards in pre-hospital settings as those in an in-hospital setting [13]. It is important to note that these guidelines were developed for the UK. The author specifically mentions that their guidelines differ significantly from those in Scandinavia, particularly due to variances in infrastructure and airway management providers [13].

The Swedish Society of Anesthesiology and Intensive care (SFAI) has developed an algorithm for difficult airway management that is widely utilized by anesthesiologist working in Sweden [14]. However, it is important to note that the resources, equipment, and environment in an in-hospital setting cannot be directly compared to those in a pre-hospital setting [14]. In the pre-hospital setting, resources and equipment are limited, varied, and the environment may be unsecure [15]. Therefore, there is a need for an alternative airway management algorithm specifically tailored to the pre-hospital setting. Currently, there is no national algorithm available for pre-hospital airway management. Practical guidelines exist for pre-hospital airway management, offering basic recommendations based on the provider’s level of training. However, there is no algorithm akin to the one developed by SFAI for difficult airway management in hospitals, which is well-established in Sweden. The physician-staffed teams operate across larger areas than individual EMS districts, which implies that a common national algorithm would facilitate collaboration between physician- staffed teams and local EMS. Given the utilization of several different local algorithms across various pre-hospital units in Sweden, and the predominantly individual or small-group nature of pre-hospital work, a consensus study was chosen to establish a unified approach to airway management. The aim of this study is to establish a consensus on how to manage difficult airways in the pre-hospital setting in Sweden for providers with advanced training.

## Methods

### Design

A two-round Delphi study was conducted to seek consensus among experts in pre-hospital emergency medicine in Sweden. The objective of this study was to establish consensus, based on that consensus, design an algorithm for managing difficult pre-hospital airways in Sweden.

The methodology involved gathering expertise on the subject matter from a group of experts through a questionnaire, which included a combination of qualitative and quantitative questions. Notably, the experts did not have direct contact or interaction with each other. After the initial round, the survey results were gathered and the panel of experts had access to the compiled answers. Subsequently, a new questionnaire was sent to the panel, which included complementary questions as needed. During this stage, the experts had the opportunity to reconsider their opinions based on the emerging perspectives shared by fellow panel members. The ultimate objective was to arrive at a united consensus on the subject, and the process continued until consensus was achieved.

### Participants

The expert panel consisted of physicians and nurse anesthetists working in Sweden in a pre-hospital setting. At the time of inclusion, there were 11 pre-hospital units in Sweden. Among these, 8 units were staffed with physicians, 2 units had nurse anesthetists staffing as standard but the option to bring a physician if needed, and 1 unit was solely staffed with a nurse.

The physicians were selected from the members of SFLPA. Permission to contact the members was obtained from SFLPA, and a membership list was provided, including the name, workplace, employment, and email address of each member (*n* = 67). All anesthesiology consultants and nurse anesthetists who works in pre-hospital setting in Sweden at the time of inclusion had the opportunity to participate in the study. Residents were excluded (*n* = 11), resulting in a panel of only consultants (*n* = 56). Participants were also included through the unit managers and/or medical directors at all 10 pre-hospital units in Sweden staffed with physicians. Physicians from all units were invited to participate in the study.

Nurses were recruited through the unit manager for nurses from two units: one helicopter and one rapid reaction car. The helicopter unit is operated by a nurse without physicians present. These units were selected for inclusion because their nurses are occasionally required to make independent decisions regarding airway management and secure the airway autonomously, for example, through ETI. The rapid reaction car unit comprises a nurse and an anesthesiologist. Nurses from this unit were included because there are instances where the physician-nurse team may be separated, and the nurse may need to independently make decisions and perform intubations. The exact number of potential participants from these units was unknown.

We assumed that some physicians were contacted both through the membership list of SFLPA and through unit managers and/or medical directors at each unit. Since the questionnaire was forwarded via email by unit managers and/or medical directors, the specific names of potential participants were not available. As a result, we were unable to obtain information on whether the potential participants were members of SFLPA or not. The participants were informed to only answer the questionnaire once, even if they received it through both their unit manager and/or medical director at each unit, as well as through the membership list of SFLPA.

All potential participants received a letter of invitation, which included an information sheet about the study and a link to the online questionnaire. This invitation was sent to them via email. They were informed that their participation in the study was voluntary, and there was no requirement to participate in the second round.

Experts were defined as consultant physicians or nurse anesthetists working in a prehospital setting in Sweden.

### Questionnaire

The questionnaire was designed using the difficult airway management algorithm from Swedish Society for Anesthesia and Intensive Care Medicine (SFAI). Each area of airway management, including decision to secure the airway, airway assessment, preparation, choice of method, optimization of airway, airway management, and choice of drugs, was organized into an Excel spreadsheet (Microsoft Corporation, Redmond, WA, USA). Based on the SFAI airway management algorithm, statements were developed for each area. These statements were then analyzed to determine their applicability in a pre-hospital setting. Statements that were deemed not applicable in a pre-hospital setting were subsequently removed. Questions were then formulated based on the remaining statements, focusing on clear differences in airway management between the pre-hospital and in-hospital settings. Additionally, questions were formulated to address various problem areas in pre-hospital airway management. The questionnaire used was developed specifically for this study and has not been previously published elsewhere. No pilot was conducted before the questionnaire was sent to the participants. The steering committee did not participate in the questionnaire. It was conducted in Swedish and was not offered in any other language. No background information was provided to the participants to avoid preconceived notions.

### First round

The participants were initially asked to provide demographic data. Number of years of experience were divided into groups (0–3, 3–5, 5–10, 10–20, 20–30 and > 30 years of experience). Following that, they were presented with questions pertaining to pre-hospital airway management and how they approach various situations, problems, and tasks in a pre-hospital setting. The questionnaire included different types of questions and statements for participants to assess (Appendix 1). In cases where there was a statement (a total of 19), participants were requested to rate their opinion on a 5-point scale, ranging from ‘1—Totally agree’ to ‘5—Totally disagree’. Optional free-text boxes were included with each question, allowing participants to provide additional comments to accompany their answers. These comments were not shared with other participants but were instead utilized to refine the statements for the second round of the questionnaire.

After the completion of the first round, the research team analyzed and summarized the responses from the questionnaire. Statements were then formulated based on the areas where a consensus was reached among the participants. In cases where there was no consensus, statements were developed by considering both the answers and comments provided by the participants.

### Second round

In the second round, the participants were contacted to complete a new questionnaire with 17 statements (Appendix 2). No link was established between the rounds, so the answers from the first round could not be linked to the answers in the second round. The results of the first round were summarized and shared with the participants. Demographic data, such as the number of respondents, the distribution across occupational categories, and the median experience of the participant group, were shared. No personally identifiable information about the participants was disclosed. The optional free-text comments from the first round were not shared with the participants. Instead, the statements formed based on the first round were presented to the participants in the second round. They were then asked to indicate their agreement or disagreement with these statements. A 3-point scale was used for rating the statements, with options including ‘1—Totally agree’, ‘2—Partially agree’, and ‘3—Totally disagree’. Prior to rating the statements, participants received a compilation of answers from the other participants in the first round. After each statement where participants had graded the statement, they were provided with an opportunity to leave additional comments in free text boxes. In these comments, participants were instructed to share their thoughts about the statements. This allowed participants to express their opinions about the statement, such as agreeing with parts of it, providing suggestions for changes, or sharing similar thoughts.

After the second round, a graphic airway management algorithm for a pre-hospital setting was designed. In conjunction with the graphic algorithm, a textual version was created based on the study.

### Consensus definition

According to Mubarak et al. [16], achieving 100% consensus among experts is rare. They suggest establishing an arbitrary percentage prior to conducting the study. In line with this, we have set the arbitrary percentage for consensus among experts at 70% agreement.

### Data collection

The digital questionnaire was administered using the online survey system Survio (Brno, Czech Republic). To prevent bias, participant anonymity was maintained throughout the data collection and analysis process. Additionally, the answers were pseudonymized for the research team. Demographic data were only accessible to the research group and could be linked to participants’ responses. The first round of the questionnaire was sent to participants on December 4, 2021, and was active until December 31, 2021. The second round was sent on April 21, 2022, and was active until May 15, 2022. Participants were reminded once per round via e-mail to submit their answers. The number of consensus steps or stopping criteria was not defined before the study. Instead, it was terminated when consensus was reached in a sufficiently large, yet undefined, proportion of statements.

## Results

In the first round, participants provided information on their demographic data (Table 1) as well as their workplace and experience in advanced airway intervention (Table 2). There was a wide range of experience among the participants, with varying years of experience in both anesthesia and the pre-hospital field. Experience levels ranged from 0–3 years to over 30 years in both areas.

**Table 1:** Demographic data of participants

**Demographic**	**Detail**	**Number (%)**
Gender	Male	58 (84)
	Female	11 (16)
Profession	Physician	51 (74)
	Nurse	18 (26)
	**Median group**	**Number in median group (%)**
Number of years of experience in anesthesia	10 to 20 years	27 (39)
Number of years of experience in pre-hospital field	5 to 10 years	15 (22)

**Table 2:** Workplace and numbers of advanced airway intervention

Topic	Detail	Number (%)
Workplace in the pre-hospital field (physicians). Possibility to work in several workplaces	Rapid reaction car	24 (47)
	Helicopter	35 (69)
	Airplane	9 (18)
	Other	3 (6)
Workplace in the pre-hospital (nurses) Possibility to work in several workplaces	Rapid reaction car	13 (72)
	Helicopter	9 (50)
	Airplane	-
	Other	1 (6)
The number of advanced pre-hospital airway interventions performed per year	0 to 10	19 (27)
	10 to 25	28 (41)
	25 to 50	20 (29)
	Over 50	2 (3)

### First round

In the first round of questions, a total of 74 nurses and physicians completed the survey. Among them, 51 were anesthesia consultants and 18 were nurse anesthetists. However, 2 emergency physicians, 1 physician without a specialist certificate, and 2 ambulance nurses were excluded from the analysis.

In the first round, there were some statements where the participants had varying opinions, resulting in disagreements (Table 3). Table 3 displays statements where participants held the most divergent opinions. All statements can be found in Appendix 1. These statements were carefully reworked for the second round based on the valuable feedback and comments provided by the participants. The aim was to refine and improve the statements to ensure greater clarity and alignment of opinions.

**Table 3:** First round, examples of statements that were reworked

Statement	Totally agree	Partially agree	Neither agree/disagree	Partially disagree	Totally disagree
In situations where I anticipate a difficult airway, which would typically be managed pre-hospital, I opt to delay securing a safe airway to a greater extent and prioritize doing so upon arrival at the hospital	*n* = 10 (14%)	*n* = 29 (40%)	*n* = 7 (10%)	*n* = 12 (16%)	*n* = 15 (21%)
	**Example of comments:** "Depending on the pathology" "Depending on the time to hospital" "Anatomical and physiological patient factors as well as expected clinical course, environmental factors, transport times etc. are decisive for decision"				
In the case of an expected difficult airway, I consider LMA as an alternative to ETI	*n* = 29 (39%)	*n* = 22 (30%)	*n* = 3 (4%)	*n* = 12 (16%)	*n* = 8 (11%)
	**Example of comments:** "LMA as a backup in case of failed intubation. Not as an primary plan" "As rescue or bridge to intubation" "If excepted, I rather see video laryngoscopy as an option. LMA rather in case of unexpected"				
I consider it important to have a high-flow nasal oxygen cannula during ETI in a pre-hospital setting	*n* = 12 (16%)	*n* = 27 (37%)	*n* = 16 (22%)	*n* = 8 (11%)	*n* = 11 (15%)
	**Example of comments:** "Depending on which type of patient and which time factors you work with" "Only where I expect a difficult airway and/or risk of severe desaturation during the intubation period" "It depend on the availability of oxygen"				
If I don’t succeed in securing the airway with ETI after two attempts, I allow another team member to make an attempt	*n* = 15 (20%)	*n* = 23 (31%)	*n* = 6 (8%)	*n* = 11 (15%)	*n* = 19 (26%)
	**Example of comments:** "Depending on the situation and which competence my colleague has" "Depending on the competence in the team but usually not" "The rest of the team usually do not have that competence"				

There were 33% (*n* = 17) respondents who indicated that no structured airway algorithm was used at the pre-hospital unit where they work. Among participants who utilized video laryngoscopy as the primary method during ETI, 67% (*n* = 35) used a Macintosh blade, while 25% (*n* = 13) used a curved blade. Additionally, 10% (*n* = 13) reported that they did not use a video laryngoscope as the primary method, opting instead for a regular Macintosh blade. Among the respondents, 67% (*n* = 35) considered that two endotracheal intubation (ETI) attempts were reasonable, while 31% (*n* = 16) considered three attempts to be reasonable before changing the method. Additionally, 100% (*n* = 52) of the participants reported using end-tidal carbon dioxide (etCO2) to confirm the correct position of the endotracheal tube, either as the sole method or in conjunction with other methods. Among the respondents, 42% (*n* = 22) totally agreed, 27% (*n* = 14) partially agreed, 8% (*n* = 4) neither agreed nor disagreed, 8% (*n* = 4) partially disagreed, and 8% (*n* = 4) totally disagreed that there is a benefit in having an airway management algorithm adapted for a pre-hospital setting.

### Second round

A total of 37 participants completed the second round.

**Fig. 1:**
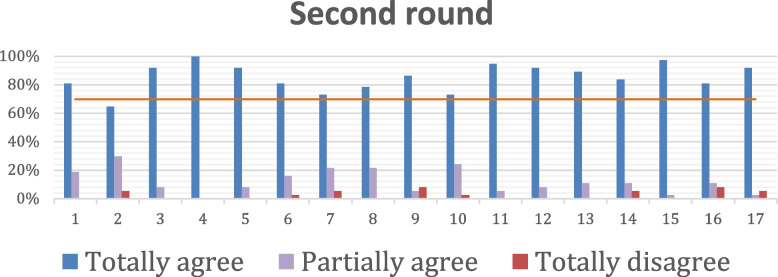
Percentage of each answer on all statements (1 to 17) in second round with consensus > 70%

Consensus for the statements was achieved after two rounds for all the 17 statements except one (Fig. 1). The statements (*n* = 17) were further catego rized into those with a high percentage of consensus (> 90% answered “totally agree”) (Table 4), intermediate percentage of consensus (80–90% answered “totally agree”) (Table 5) and those with lower percentage of consensus (< 80% answered “totally agree”) (Table 6).

**Table 4:** Second round, statements with high percentage consensus (> 90% answered "totally agree")

Statements	Totally agree	Partially agree	Totally disagree
Having a clear plan regarding drugs and potential respiratory difficulties should either be standardized within the respective unit or established before a patient undergoes intubated, involve everyone in the team	*n* = 34 (92%)	*n* = 3 (8%)	*n* = 0 (0%)
Ensure that all necessary equipment for airway management is either prepared prior to anesthesia or readily available and well-organized. This way, the entire team will be aware of the equipment’s location and can promptly retrieve it when needed	*n* = 37 (100%)	*n* = 0 (0%)	*n* = 0 (0%)
It is important and patient-safe to have an algorithm for pre-hospital airway management, and there are benefits to using an adapted algorithm for pre-hospital work. It is also crucial that the algorithm is simple and does not complicate the work further. While an algorithm should serve as the foundation of the work, it is important to allow flexibility based on experience, knowledge, and the specific situation	*n* = 34 (92%)	*n* = 3 (8%)	*n* = 0 (0%)
If the situation allows and there are team members experienced in anesthesia, it may be advisable to assign them the task of performing an intubation attempt. In the event that the situation escalates to a ‘can’t ventilate, can’t oxygenate’ scenario, a surgical airway becomes the final option and should be conducted by the healthcare professional with the highest level of competence for this procedure. The chosen method should align with the individual’s familiarity and be carried out in the simplest manner possible	*n* = 35 (95%)	*n* = 2 (5%)	*n* = 0 (0%)
While there may be various inventive tricks and methods in airway management, it is generally recommended to rely on established techniques and utilize the simplest possible algorithm that you are comfortable with. It is important to avoid complicating or delaying airway management with advanced methods	*n* = 34 (92%)	*n* = 3 (8%)	*n* = 0 (0%)
To optimize airway management and ensure deep relaxation without the need for repeated interventions, it is important to properly relax the patient. If necessary, anesthesia can be deepened to provide comfort to the patient. It is crucial to consider the patient’s circulation, pathology, and adapt the choice of drugs when dosing the anesthesia	*n* = 36 (97%)	*n* = 1 (3%)	*n* = 0 (0%)
If possible, it is advisable to enlist the assistance of other on-site personnel such as ambulance crews, firefighters, police officers, etc., to handle monitoring and provide support. This allows individuals with anesthesia experience to concentrate on airway management and administering drugs. Clear instructions should be provided to these individuals, based on their competence, regarding their assigned tasks and what changes in vital parameters should be reported during monitoring. If these personnel possess the necessary competence, they can also assist with tasks such as cervical spine stabilization, suction, administering infusions, and more	*n* = 34 (92%)	*n* = 1 (3%)	*n* = 2 (5%)

**Table 5:** Second round, statements with intermediate percentage consensus (80–90% answered "totally agree")

Statements	Totally agree	Partially agree	Totally disagree
If the airway is assessed as difficult, the best possible conditions based on the situation and location should be obtained. In cases where the pathology does not absolutely require a secure airway, one should wait to secure the airway until in a location where more resources and optimal conditions can be provided. Also, consideration should be given to transport time and how this will be carried out when deciding to secure the airway or not at the scene of the injury	*n* = 30 (81%)	*n* = 7 (19%)	*n* = 0 (0%)
After the decision for ETI, a quick airway assessment should be conducted to formulate a plan for its management and how potential issues will be handled. Often, a visual assessment is sufficient, but if one assesses that problems may arise with airway management or if the situation and the patient’s condition permit, a more thorough examination of the airway should be performed	*n* = 30 (81%)	*n* = 6 (16%)	*n* = 1 (3%)
When time allows, it may be considered to have a high-flow nasal oxygen cannula during airway management to optimize oxygenation and gain time in case of difficulties with airway management. Both the time factor and the availability of oxygen affect this possibility. However, this should not replace or compromise the usual preoxygenation if it becomes difficult to maintain a tight seal. In these cases, the mask can be prepared on the forehead/neck and inserted into the nose when preoxygenation is complete	*n* = 32 (87%)	*n* = 2 (5%)	*n* = 3 (8%)
To achieve the best possible conditions for successful airway management, if circumstances allow, the patient’s position should be optimized. This can be done by moving the patient to a location with better conditions and, if necessary, placing something under the patient’s neck if available. However, one should consider that the neck should be protected in trauma patients where spinal cord injury cannot be ruled out	*n* = 33 (89%)	*n* = 4 (11%)	*n* = 0 (0%)
To confirm that the tube has entered the trachea, carbon dioxide measurement should primarily be used. Additionally, visual confirmation of the tube passing the vocal cords, listening over the stomach, auscultation of the lungs, condensation in the tube, and chest movement can be used as a supplement to carbon dioxide measurement, which should be the gold standard	*n* = 31 (84%)	*n* = 4 (11%)	*n* = 2 (5%)
Patients for whom the decision to intubate is made pre-hospitally should have a clear indication that justifies the decision to intubate. Therefore, the decision to perform ETI and later encountering problems with airway management seldom involves waking up the patient, except in isolated cases where the situation allows it. In these situations, the decision to initially perform ETI the patient should have been questioned	*n* = 30 (81%)	*n* = 4 (11%)	*n* = 3 (8%)

**Table 6:** Second round, statements with low percentage consensus (< 80% answered "totally agree")

Statements	Totally agree	Partially agree	Totally disagree
If the decision to intubate has been made due to the pathology or circumstances necessitating it, the assessment that the airway is difficult should not impact the decision to proceed with intubation. Nonetheless, it is crucial to engage in meticulous preparation and develop a comprehensive plan for airway management	*n* = 24 (65%)	*n* = 11 (30%)	*n* = 2 (5%)
A SAD can serve as an alternative when a difficult airway is anticipated. However, in cases where airway protection against aspiration is necessary, the first attempt should be ETI. In situations where aspiration is not a concern, a SAD can be considered as the initial choice if a difficult airway is expected. If ETI is unsuccessful, the subsequent option should involve attempting insertion of a SAD to ensure patient ventilation	*n* = 27 (73%)	*n* = 8 (22%)	*n* = 2 (5%)
When performing ETI, video laryngoscopes equipped with Macintosh blades may be the preferred choice, if available. However, in situations involving vomiting, bleeding, or direct sunlight, it may be advisable to primarily use a regular laryngoscope to avoid the risk of obtaining a compromised image. Nevertheless, it is worth considering the use of a video laryngoscope, as it can function similarly to a regular laryngoscope when a Macintosh blade is employed	*n* = 29 (78%)	*n* = 8 (22%)	*n* = 0 (0%)
A maximum of two attempts at ETI should be made, conducted by the individual with the highest level of competence. In cases where the patient’s condition or the situation necessitates it, a third attempt may be considered. However, it is important to carefully assess the likelihood of success and explore alternative methods of ventilation (e.g., mask ventilation, SAD) before proceeding with the third attempt. Additionally, it is crucial to evaluate and adjust the procedure accordingly based on the preceding attempts	*n* = 27 (73%)	*n* = 9 (24%)	*n* = 1 (3%)

**Fig. 2:**
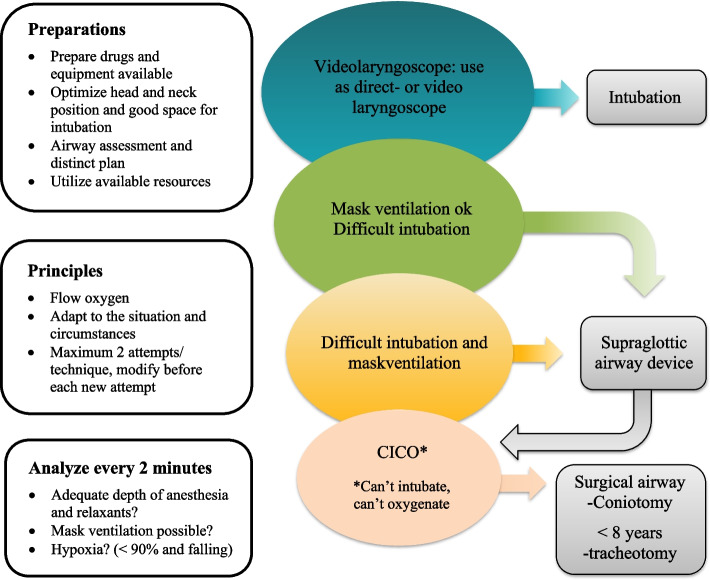
Pre-hospital difficult airway algorithm

Based on the statements where were consensus was reached, a pre-hospital difficult airway algorithm was formed (Fig. 2).

## Discussion

This study capitalizes on the extensive practical experience of participants in prehospital airway management, ensuring the relevance and applicability of the findings. Through a Delphi study approach, participants shared their insights, fostering collaborative decision-making to reach a consensus on the design of an optimal airway algorithm for prehospital settings. Remarkably, this study successfully achieved consensus on 16 out of 17 statements regarding the management of airways in pre-hospital settings.

The expert panel unanimously recognized the advantages of implementing a standardized airway management algorithm tailored to the context of pre-hospital operations in Sweden, to ensure a consistent approach to airway management. The findings of this study indicate the existence of several local algorithms across various pre-hospital units in Sweden. However, it is recognized that an algorithm should ideally be standardized and easily reproducible across all pre-hospital organizations [13]. This underscores the significance of having a standardized algorithm to ensure consistency among providers across different pre-hospital units in airway management. Standardization ensures that all personnel follow the same approach and utilize the same algorithm. With a standardized algorithm in place, individuals working in any pre-hospital setting will be familiar with the procedure, promoting uniformity in care delivery.

Furthermore, the findings of this study emphasize the importance of flexibility in adapting management strategies to suit the unique circumstances of each situation and patient. Pre-hospital work varies from case to case, necessitating the ability to tailor management approaches accordingly. It is important to recognize that the algorithm developed in this study should be regarded as a recommendation rather than strict instructions for management.

The algorithm we developed differs from many other algorithms designed for in-hospital use. We began with SFAI’s difficult intubation algorithm when formulating statements, and finally, the algorithm was refined. In contrast to SFAI’s algorithm, videolaryngoscopy is the recommended primary choice, either for direct video use or as a regular laryngoscope. The use of fiberoptic intubation is not feasible in pre-hospital settings due to its limited availability. In the new algorithm, emphasis is placed on preparing drugs and having equipment readily available, optimizing head/neck positioning, ensuring ample space for intubation, establishing a clear plan before starting, utilizing available resources, and adapting management to the situation. While these are elements typically considered in airway management situations, they assume greater importance in the prehospital setting and therefore warrant special emphasis. The option of waking the patient is seldom practical and has been removed from the algorithm. Additionally, the algorithm recommends a maximum of two attempts for each technique instead of three, with a modification of the technique before each new attempt.

The introduction of a new standard operating procedure (SOP) for pre-hospital anesthesia in the UK in 2012 aimed to improve the success rates of ETI. This initiative resulted in no failed ETI during the study period, as reported in study [17]. Similar studies have also demonstrated the advantages of implementing a SOP, such as higher rates of first-pass success and reduced instances of failed ETI [18]. These findings provide valuable support for the evidence-based implementation of an airway management algorithm in a pre-hospital setting.

In a study conducted in the Nordic countries, the implementation of a pre-intubation checklist was examined to determine its impact on pre-hospital ETI among experienced anesthesiologists [19]. Although the overall success rate for ETI did not show a significant difference (99.1% vs. 99.4%), the first attempt ETI success rate increased from 86.2% to 96.6% after the checklist was implemented [19]. Additionally, the study found a decrease in complications associated with ETI [19]. By having a standardized approach, the chances of achieving a successful first-time ETI will be greater, and the time spent on the scene will be minimized. This, in the long term, would improve healthcare for the patient.

Failure to intubate a difficult airway is very rare when using an algorithm, previous studies have shown rates as low as 0.1% when the algorithm is implemented by trained operators [20]. Interestingly, in our study, we found that one out of three participants did not have any airway management algorithm in their pre-hospital unit. Another one out of three participants used a customized algorithm from the SFAI that was originally designed for the in-hospital environment. The remaining participants had a local algorithm specific to their unit. Numerous previous studies have highlighted the advantages of implementing a standardized approach, which includes using algorithms and checklists, when administering anesthesia and managing airways in a pre-hospital setting [11, 18, 20, 21]. The findings of the present study align with these previous studies, demonstrating a consensus that an algorithm is crucial for both successful airway management and ensuring patient safety. It is essential to customize the algorithm specifically for pre-hospital situations, taking into consideration the unique challenges and factors that exist in that setting. By adopting a tailored algorithm, healthcare providers can optimize their ability to effectively handle airways and prioritize patient safety in the pre-hospital environment.

When designing an algorithm for pre-hospital airway management, it is essential to ensure that it meets the same safety standards as those used in the in-hospital anesthesia setting. Reproducibility is also crucial, as it allows for consistency and uniformity in the application of the algorithm across different healthcare providers and situations. Participants in the study emphasized the importance of simplicity in the algorithm, as it should not unnecessarily complicate their work. This aligns with a previous study that demonstrated how a simple and concise algorithm can effectively reduce errors caused by human factors and enhance patient safety [22]. By keeping the algorithm straightforward and relatively short, healthcare providers can easily follow and implement it, thereby maximizing its benefits in improving patient safety during pre-hospital airway management.

The participants stated that while algorithms should provide the foundation for their work, there should also be flexibility to adapt one’s approach based on experience, knowledge, and the specific situation. It’s worth noting that using algorithms can have its drawbacks as well. For instance, when checklists are employed, it has been observed that the time spent on the scene of injury tends to be longer [19]. This, unfortunately, leads to delays in the patient’s arrival at the hospital and, ultimately, may impact the patient’s chances of survival [19]. The findings also highlight that each pre-hospital site where ETI is performed is unique, posing challenges in developing a standardized algorithm that fits every situation optimally [19]. This aligns with the results of our study. A previous study has addressed that when an algorithm not specifically developed for pre-hospital care is utilized, it can lead to issues and deficiencies in equipment [23].

Recent knowledge indicates that utilizing a limited range of equipment is advantageous when managing a difficult airway in an emergency situation [24]. This approach offers several benefits, including a clearer overview, reduced complexity in mastering techniques, and quicker decision-making and access [24]. This mindset is also applicable in a pre-hospital setting. The participants reached a consensus that all equipment required for airway management should either be readily available before anesthesia or easily accessible and well-organized, ensuring that every team member knows the equipment’s location and can quickly retrieve it if needed. There was also agreement on the importance of having a clear plan for administering drugs, either through standardization or preparation prior to patient intubation. It was emphasized that all team members involved in airway management should be actively engaged in the planning process.

Airway management in a pre-hospital setting presents unique challenges compared to in-hospital airway management. The pre-hospital environment is often unsafe, stressful, and characterized by limited resources. These factors can significantly impact the success rate of pre-hospital ETI [20]. The participants in our study emphasize the importance of ensuring safe and effective airway management in the pre-hospital setting, considering it a high-risk procedure. This may explain why there was no agreement on the timing of performing ETI. However, when faced with a decision to perform ETI and an airway assessment indicating a potential risk for a difficult airway, 64.9% of participants totally agreed that the potential difficulty should not influence the decision to perform ETI. This was the only statement in which the panel did not reach a consensus. The limited resources and equipment available in a pre-hospital setting could be the underlying reason for this divergence of opinions. Unlike an in-hospital setting, the options for managing airways are significantly constrained in pre-hospital settings. This constraint can potentially lead to challenges and ultimately result in patient injuries. Some participants highlighted that in certain cases where ETI would be the preferred course of action in an in-hospital setting, the appropriate decision in a pre-hospital setting could be to refrain from performing ETI. The findings of the present study also indicate that the decision to perform ETI or not is based on a comprehensive assessment. Additionally, there may be situations where the need for ETI arises from the necessity of transporting the patient to a hospital rather than solely from concerns related to an unsafe airway.

There was a consensus among the participants in our study that the most experienced individuals should perform the ETI, with a maximum of two attempts. The reason for this can be surmised to be a higher success rate. This is confirmed by previous studies, which indicate that an experienced performer has a higher success rate, especially among anesthetists and physicians compared to non-physicians [25]. In a meta-analysis of 1070 studies, it was concluded that ‘physicians experience significantly fewer pre-hospital ETI failures overall than non-physicians’ [25]. A significantly higher success rate was observed among physicians in pre-hospital settings compared to non-physicians, with median success rates of 99.1% vs. 84.9%, respectively [25]. This discrepancy was attributed to the physicians’ training and greater experience [25].

In a large observational study of 7256 patients who required advanced airway management between 1991 and 2012 in the UK, it was shown that ETI performed by non-anesthetists failed in 0.9% of cases, compared to 0.4% among anesthetists [23]. The study demonstrated significantly better first-pass ETI rates for anesthesiologists and consultant emergency physicians compared to emergency medicine trainees [24]. Additionally, the quality of laryngeal views was superior in doctors with an anesthetic background [24].

In Scandinavia, the success rates are similar [26]. The overall success rate was 98.7%, with anesthetists achieving a success rate of 99.0%, while nurse anesthetists achieved a success rate of 97.6% [26]. SSAI recommends that ETI should only be performed by anesthesiologists [27]. Other physicians, paramedics, and EMS personnel are advised to use assisted mask ventilation in combination with the lateral trauma recovery position [27]. During cardiopulmonary resuscitation, a SAD should be used by non-anesthesiologists [27].

The participants in our study also reached the conclusion that in a ‘can’t ventilate, can’t oxygenate’ situation, the same approach should be adopted: the most experienced individual in the procedure should perform a surgical airway.

The SSAI has published a clinical practice guideline for pre-hospital airway management, stating that a videolaryngoscope should be employed when difficult direct laryngoscopy is expected or when direct laryngoscopy attempts fail [12]. A prior study corroborates this, as it investigated 152 ETIs performed by anesthesiologists on patients with either anticipated or unanticipated difficult airways, and found no instances of failed ETIs [28]. Additionally, studies have demonstrated that the first-pass success rate is higher when using a videolaryngoscope compared to a direct laryngoscope [26]. These findings align with the opinions of our study participants. The results of our current study suggest that a videolaryngoscope with a Macintosh blade could be employed in all ETIs. It can serve as a direct laryngoscope when preferred, and in case challenges arise, the transition to video-assisted intubation is straightforward and accessible. The use of an ordinary laryngoscope should only be the first choice in situations where the videolaryngoscope presents a disadvantage, such as when there is direct sunlight, ongoing vomiting, or bleeding.

The subsequent phase involves the validation of the algorithm.

### Limitations

This study possesses certain limitations that necessitate acknowledgment. Some statements in the questionnaire are lengthy and may benefit from being divided into smaller, more concise statements. This could lead to a more consistent perception of the content among participants. In the second round of data collection, only half of the participants from the initial round participated. However, it is reasonable to infer that those who did not continue into the second round had likely already conveyed their opinions during the first round. Unfortunately, during the second round, we did not gather information regarding the participants’ professions, rendering the occupational backgrounds of dropouts unknown. Hence, we lack data regarding whether participants in the second round included only physicians or also nurses. Notably, in the first round, no distinctions emerged in the responses between physicians and nurses. Consequently, we may assume that noteworthy variances between the answers of physicians and nurses did not materialize in the second round.

The selection of the arbitrary consensus threshold of 70%, while rooted in precedent studies, remains open to discussion and potential adjustment.

## Conclusion

The capacity to adapt the approach to airway management based on specific pre-hospital circumstances is crucial. It holds significance to establish a uniform framework that is applicable across various airway management scenarios. Consequently, the airway management algorithm that has been devised should be regarded as a recommendation, allowing for flexibility rather than being interpreted as a rigid course of action.

This represents the inaugural nationwide algorithm for airway management designed exclusively for pre-hospital operations in Sweden. The algorithm is the result of a consensus reached by experts in pre-hospital care.

### Supplementary Information

Supplementary Material 1.

Supplementary Material 2.

## Data Availability

Data information is available from the corresponding author on reasonable request.

## Abbreviations

EMS: Emergency medical service
ETI: Endotracheal intubation
NAP4: Fourth National Audit Projects
SAD: Supraglottic airway device
SFAI: Swedish Society for Anesthesia and Intensive Care Medicine
SFLPA: Swedish Association of Physicians in Prehospital Emergency Medicine
SOP: Standard operating procedure
SSAI: Scandinavian Society of Anaesthesiology and Intensive Care Medicine
