# Supplementary material for: Swedish consensus regarding difficult pre-hospital airway management: a Delphi study
Source: BMC Emerg Med. 2024 May 27;24:88. doi: 10.1186/s12873-024-01013-x (PMC11129497; doi:10.1186/s12873-024-01013-x)
Supplement: Supplementary file 1 — Supplementary Material 1. [file 12873_2024_1013_MOESM1_ESM.pdf]

Prehospital airway management

Thank you for taking the time to fill out this survey!

There are a total of 35 questions that take 5-10 minutes to answer.

The questions should be answered based on your work in a pre-hospital setting, not how you work in other workplaces.

1 Occupational category?

Question instructions:

- [ ] Anaesthesiologist
- [ ] Anesthesia nurse
- [ ] Other (specify) __________

2 How many years have you been clinically active as a specialist doctor/specialist nurse in anesthesia?

Question instructions:

- [ ] 0-3 years
- [ ] 3-5 years
- [ ] 5-10 years
- [ ] 10-20 years
- [ ] 20-30 years
- [ ] More than 30 years
- [ ] Not specialist in anesthesia
- [ ] Comments __________

3 How many years have you worked pre-hospitally as a doctor/nurse?

Question instructions:

- [ ] 0-3 years
- [ ] 3-5 years
- [ ] 5-10 years
- [ ] 10-20 years
- [ ] 20-30 years
- [ ] More than 30 years
- [ ] What percentage do you estimate that you have worked on average per year pre-hospitally (100% corresponds to full-time)? __________

4 Gender

- ◯ Male
- ◯ Female
- ◯ Do not want to disclose

5 Age

[ ]

6 Workplace in the pre-hospital field

Question instructions: *Choose one or more answers*

- ☐ Rapid reaction car
- ☐ Helicopter
- ☐ Airplane
- ☐ Other (specify) [ ]

7 Number of advanced pre-hospital airway interventions (including endotracheal intubation, laryngeal mask airway etc.) performed per year

Question instructions:

- ☐ 0-10
- ☐ 10-25
- ☐ 25-50
- ☐ More than 50
- ☐ Comments [ ]

8 In situations where I anticipate a difficult airway, which would typically be managed pre-hospital, I opt to delay securing a safe airway to a greater extent and prioritize doing so upon arrival at the hospital

Question instructions:

- ☐ Totally agree
- ☐ Partially agree
- ☐ Neither agree/disagree
- ☐ Partially disagree
- ☐ Totally disagree
- ☐ Comments? [ ]

9 In the case of an expected difficult airway, I consider laryngeal mask airway as an alternative to endotracheal intubation

Question instructions:

- [ ] Totally agree
- [ ] Partially agree
- [ ] Neither agree/disagree
- [ ] Partially disagree
- [ ] Totally disagree
- [ ] Comments? ______

10 Do you perform an airway assessment before endotracheal intubation on a patient where you have ALREADY decided on endotracheal intubation?

Question instructions:

- [ ] Yes
- [ ] No
- [ ] Comments ______

11 Do you make a thorough assessment of the airway before endotracheal intubation (e.g., open the mouth, flex the neck, etc.) or a visual assessment?

Question instructions:

- [ ] Thorough assessment
- [ ] Visual assessment
- [ ] Comments? ______

12 Is a structured airway algorithm used in the pre-hospital unit you work in, and if so, which one(s) are used?

Question instructions: *Choose one or more answers*

- [ ] SFAI's airway algorithm
- [ ] Local algorithm
- [ ] No
- [ ] Other (specify)? ______

13 I consider it important to have a high-flow nasal oxygen cannula during endotracheal intubation in a pre-hospital setting

Question instructions:

- [ ] Totally agree
- [ ] Partially agree
- [ ] Neither agree/disagree
- [ ] Partially disagree
- [ ] Totally disagree
- [ ] Comments?

14 I consider it important to use videolaryngoscopy as the first method in endotracheal intubation in a pre-hospital setting

Question instructions:

- [ ] Totally agree
- [ ] Partially agree
- [ ] Neither agree/disagree
- [ ] Partially disagree
- [ ] Totally disagree
- [ ] Comments?

15 If you use videolaryngoscopy as the first method for laryngoscopy, which blade do you use?

Question instructions:

- [ ] Macintosh blade
- [ ] Hyper curved
- [ ] Use regular laryngoscope first
- [ ] Comments?

16 I consider it important to have a clear plan with drug dosage with my assistant before an endotracheal intubation

Question instructions:

- [ ] Totally agree
- [ ] Partially agree
- [ ] Neither agree/disagree
- [ ] Partially disagree
- [ ] Totally disagree
- [ ] Comments?

17 I believe it is important to have a clear and communicated plan for airway management if the primary plan does not work

Question instructions:

- [ ] Totally agree
- [ ] Partially agree
- [ ] Neither agree/disagree
- [ ] Partially disagree
- [ ] Totally disagree
- [ ] Comments?

18 I believe it is important to prepare additional equipment before endotracheal intubation to have it readily available in case of difficulties (Bougie, laryngeal mask airway, other tubes)

Question instructions:

- [ ] Totally agree
- [ ] Partially agree
- [ ] Neither agree/disagree
- [ ] Partially disagree
- [ ] Totally disagree
- [ ] Comments?

19 If mask ventilation with possible other aids (e.g., oropharyngeal airway) is difficult, I try using a laryngeal mask airway in the first instance instead of endotracheal intubation

Question instructions:

- ☐ Totally agree
- ☐ Partially agree
- ☐ Neither agree/disagree
- ☐ Partially disagree
- ☐ Totally disagree
- ☐ Comments? ____________

20 How many endotracheal intubation attempts do I consider reasonable before deciding to use other alternatives (e.g., laryngeal mask airway)?

Question instructions:

- ☐ 1
- ☐ 2
- ☐ 3
- ☐ 4
- ☐ >5
- ☐ Comments? ____________

21 If I don’t succeed in securing the airway with endotracheal intubation after two attempts, I allow another team member to make an attempt.

Question instructions:

- ☐ Totally agree
- ☐ Partially agree
- ☐ Neither agree/disagree
- ☐ Partially disagree
- ☐ Totally disagree
- ☐ Comments? ____________

22 I believe that if endotracheal intubation is difficult, my first action is to deepen the anesthesia instead of resorting to placing a laryngeal mask airway

Question instructions:

- [ ] Totally agree
- [ ] Partially agree
- [ ] Neither agree/disagree
- [ ] Partially disagree
- [ ] Totally disagree
- [ ] Comments?

23 I believe it is important to deepen the anesthesia and administer muscle relaxant every other minute if I am unable to perform endotracheal intubation

Question instructions:

- [ ] Totally agree
- [ ] Partially agree
- [ ] Neither agree/disagree
- [ ] Partially disagree
- [ ] Totally disagree
- [ ] Comments?

24 I believe that waking up the patient is an alternative in a pre-hospital setting if I am unable to perform endotracheal intubation

Question instructions:

- [ ] Totally agree
- [ ] Partially agree
- [ ] Neither agree/disagree
- [ ] Partially disagree
- [ ] Totally disagree
- [ ] Comments?

25 We have an adequate amount of Bridion available on our unit to completely reverse

Question instructions:

- ◯ Yes
- ◯ No
- ◯ I don't know

26 I believe it is important to optimize the position of the patient with pillows/blankets

Question instructions:

- ☐ Totally agree
- ☐ Partially agree
- ☐ Neither agree/disagree
- ☐ Partially disagree
- ☐ Totally disagree
- ☐ Comments? ______

27 I believe it is important to instruct other personnel on site (not an anesthesia nurse/anesthesiologist assisting with endotracheal intubation/medication) to monitor the patients monitoring during the airway management

Question instructions:

- ☐ Totally agree
- ☐ Partially agree
- ☐ Neither agree/disagree
- ☐ Partially disagree
- ☐ Totally disagree
- ☐ Comments? ______

28 I believe that a surgical airway is an important and realistic alternative if I cannot intubate in a pre-hospital setting

Question instructions:

- ☐ Totally agree
- ☐ Partially agree
- ☐ Neither agree/disagree
- ☐ Partially disagree
- ☐ Totally disagree
- ☐ Comments? [ ]

29 Do you use any alternative measures (creative alternatives) if you are unsuccessful with endotracheal intubation/laryngeal mask airway?

[ ]

30 I believe that capnography is the correct method to use to confirm the correct tube placement

Question instructions:

- ☐ Totally agree
- ☐ Partially agree
- ☐ Neither agree/disagree
- ☐ Partially disagree
- ☐ Totally disagree
- ☐ Comments? [ ]

31 Which methods do you use to confirm the correct tube placement?

Question instructions: *Choose one or more answers*

- ☐ Stetoscope
- ☐ etCO2
- ☐ Condensation inside the tube
- ☐ Movement of the chest
- ☐ Other method? [ ]

32 Does my airway management differ in a pre-hospital setting compared to work in a hospital setting?

Question instructions:

- ◯ Totally agree
- ◯ Partially agree
- ◯ Neither agree/disagree
- ◯ Partially disagree
- ◯ Totally disagree
- ◯ Comments? [ ]

33 I believe it is important and safe for the patient to have an algorithm that is similar in a pre-hospital setting and in the hospital?

Question instructions:

- ◯ Totally agree
- ◯ Partially agree
- ◯ Neither agree/disagree
- ◯ Partially disagree
- ◯ Totally disagree
- ◯ Comments? [ ]

34 I believe there is a benefit in having an airway algorithm adapted for pre-hospital operations

Question instructions:

- ◯ Totally agree
- ◯ Partially agree
- ◯ Neither agree/disagree
- ◯ Partially disagree
- ◯ Totally disagree
- ◯ Comments? [ ]

35 Do you have any comments or opinions you would like to convey?

[ ]

Thank you very much for your participation and your opinions!
