# Supplementary material for: Swedish consensus regarding difficult pre-hospital airway management: a Delphi study
Source: BMC Emerg Med. 2024 May 27;24:88. doi: 10.1186/s12873-024-01013-x (PMC11129497; doi:10.1186/s12873-024-01013-x)
Supplement: Supplementary file 2 — Supplementary Material 2. [file 12873_2024_1013_MOESM2_ESM.pdf]

Prehospital airway management 2

The following statements about prehospital airway management are based on responses from the first survey sent out in December 2021. We would like you to take a position on the statements by either agreeing or disagreeing. If you disagree with the statement, we ask that you provide free-text comments on what changes you would like to see for you to agree with the statement.

The number of respondents in part one was 75, including 52 specialist doctors in anesthesia, 18 anesthesia nurses, and 5 others. The majority had 5-20 years of experience as a qualified specialist doctor/anesthesia nurse.

Based on the answers, it is concluded that prehospital airway management is situation- and environment-dependent. It is challenging to create a ready-made template for how airway management should be carried out, while many are calling for standardization to lean on.

1 If the airway is assessed as difficult, the best possible conditions based on the situation and location should be obtained. In cases where the pathology does not absolutely require a secure airway, one should wait to secure the airway until in a location where more resources and optimal conditions can be provided. Also, consideration should be given to transport time and how this will be carried out when deciding to secure the airway or not at the scene of the injury.

Question instructions: *What is your stance on the statement above?*

- ◯ Totally agree
- ◯ Partially agree
- ◯ Totally disagree

2 What do you want to change?

3 If the decision to intubate has been made due to the pathology or circumstances necessitating it, the assessment that the airway is difficult should not impact the decision to proceed with endotracheal intubation. Nonetheless, it is crucial to engage in meticulous preparation and develop a comprehensive plan for airway management.

Question instructions: *What is your stance on the statement above?*

- ◯ Totally agree
- ◯ Partially agree
- ◯ Totally disagree

4 What do you want to change?

5 Having a clear plan regarding drugs and potential respiratory difficulties should either be standardized within the respective unit or established before a patient undergoes endotracheal intubation, involve everyone in the team.

Question instructions: *What is your stance on the statement above?*

- ◯ Totally agree
- ◯ Partially agree
- ◯ Totally disagree

6 What do you want to change?

7 Ensure that all necessary equipment for airway management is either prepared prior to anesthesia or readily available and well-organized. This way, the entire team will be aware of the equipment's location and can promptly retrieve it when needed.

Question instructions: *What is your stance on the statement above?*

- ◯ Totally agree
- ◯ Partially agree
- ◯ Totally disagree

8 What do you want to change?

9 It is important and patient-safe to have an algorithm for pre-hospital airway management, and there are benefits to using an adapted algorithm for pre-hospital work. It is also crucial that the algorithm is simple and does not complicate the work further. While an algorithm should serve as the foundation of the work, it is important to allow flexibility based on experience, knowledge, and the specific situation.

Question instructions: *What is your stance on the statement above?*

- ◯ Totally agree
- ◯ Partially agree
- ◯ Totally disagree

10 What do you want to change?

11 After the decision for endotracheal intubation, a quick airway assessment should be conducted to formulate a plan for its management and how potential issues will be handled. Often, a visual assessment is sufficient, but if one assesses that problems may arise with airway management or if the situation and the patient's condition permit, a more thorough examination of the airway should be performed.

Question instructions: *What is your stance on the statement above?*

- ◯ Totally agree
- ◯ Partially agree
- ◯ Totally disagree

12 What do you want to change?

13 A supraglottic airway device can serve as an alternative when a difficult airway is anticipated. However, in cases where airway protection against aspiration is necessary, the first attempt should be endotracheal intubation. In situations where aspiration is not a concern, a supraglottic airway device can be considered as the initial choice if a difficult airway is expected. If endotracheal intubation is unsuccessful, the subsequent option should involve attempting insertion of a supraglottic airway device to ensure patient ventilation.

Question instructions: *What is your stance on the statement above?*

- ◯ Totally agree
- ◯ Partially agree
- ◯ Totally disagree

14 What do you want to change?

15 When performing endotracheal intubation, video laryngoscopes equipped with Macintosh blades may be the preferred choice, if available. However, in situations involving vomiting, bleeding, or direct sunlight, it may be advisable to primarily use a regular laryngoscope to avoid the risk of obtaining a compromised image. Nevertheless, it is worth considering the use of a video laryngoscope, as it can function similarly to a regular laryngoscope when a Macintosh blade is employed.

Question instructions: *What is your stance on the statement above?*

- ◯ Totally agree
- ◯ Partially agree
- ◯ Totally disagree

16 What do you want to change?

17 When time allows, it may be considered to have a high-flow nasal oxygen cannula during airway management to optimize oxygenation and gain time in case of difficulties with airway management. Both the time factor and the availability of oxygen affect this possibility. However, this should not replace or compromise the usual preoxygenation if it becomes difficult to maintain a tight seal. In these cases, the mask can be prepared on the forehead/neck and inserted into the nose when preoxygenation is complete.

Question instructions: *What is your stance on the statement above?*

- ◯ Totally agree
- ◯ Partially agree
- ◯ Totally disagree

18 What do you want to change?

19 A maximum of two attempts at endotracheal intubation should be made, conducted by the individual with the highest level of competence. In cases where the patient's condition or the situation necessitates it, a third attempt may be considered. However, it is important to carefully assess the likelihood of success and explore alternative methods of ventilation (e.g., mask ventilation, supraglottic airway devices) before proceeding with the third attempt. Additionally, it is crucial to evaluate and adjust the procedure accordingly based on the preceding attempts.

Question instructions: *What is your stance on the statement above?*

- ◯ Totally agree
- ◯ Partially agree
- ◯ Totally disagree

20 What do you want to change?

21 If the situation allows and there are team members experienced in anesthesia, it may be advisable to assign them the task of performing an endotracheal intubation attempt. In the event that the situation escalates to a 'can't ventilate, can't oxygenate' scenario, a surgical airway becomes the final option and should be conducted by the healthcare professional with the highest level of competence for this procedure. The chosen method should align with the individual's familiarity and be carried out in the simplest manner possible.

Question instructions: *What is your stance on the statement above?*

- ◯ Totally agree
- ◯ Partially agree
- ◯ Totally disagree

22 What do you want to change?

23 While there may be various inventive tricks and methods in airway management, it is generally recommended to rely on established techniques and utilize the simplest possible algorithm that you are comfortable with. It is important to avoid complicating or delaying airway management with advanced methods.

Question instructions: *What is your stance on the statement above?*

- ◯ Totally agree
- ◯ Partially agree
- ◯ Totally disagree

24 What do you want to change?

25 To achieve the best possible conditions for successful airway management, if circumstances allow, the patient's position should be optimized. This can be done by moving the patient to a location with better conditions and, if necessary, placing something under the patient's neck if available. However, one should consider that the neck should be protected in trauma patients where spinal cord injury cannot be ruled out.

Question instructions: *What is your stance on the statement above?*

- ◯ Totally agree
- ◯ Partially agree
- ◯ Totally disagree

26 What do you want to change?

27 To confirm that the tube has entered the trachea, carbon dioxide measurement should primarily be used. Additionally, visual confirmation of the tube passing the vocal cords, listening over the stomach, auscultation of the lungs, condensation in the tube, and chest movement can be used as a supplement to carbon dioxide measurement, which should be the gold standard.

Question instructions: *What is your stance on the statement above?*

- ◯ Totally agree
- ◯ Partially agree
- ◯ Totally disagree

28 What do you want to change?

29 To optimize airway management and ensure deep relaxation without the need for repeated interventions, it is important to properly relax the patient. If necessary, anesthesia can be deepened to provide comfort to the patient. It is crucial to consider the patient's circulation, pathology, and adapt the choice of drugs when dosing the anesthesia.

Question instructions: *What is your stance on the statement above?*

- ◯ Totally agree
- ◯ Partially agree
- ◯ Totally disagree

30 What do you want to change?

31 Patients for whom the decision to intubate is made pre-hospitally should have a clear indication that justifies the decision to intubate. Therefore, the decision to perform endotracheal intubation and later encountering problems with airway management seldom involves waking up the patient, except in isolated cases where the situation allows it. In these situations, the decision to initially perform endotracheal intubation the patient should have been questioned.

Question instructions: *What is your stance on the statement above?*

- ◯ Totally agree
- ◯ Partially agree
- ◯ Totally disagree

32 What do you want to change?

33 If possible, it is advisable to enlist the assistance of other on-site personnel such as ambulance crews, firefighters, police officers, etc., to handle monitoring and provide support. This allows individuals with anesthesia experience to concentrate on airway management and administering drugs. Clear instructions should be provided to these individuals, based on their competence, regarding their assigned tasks and what changes in vital parameters should be reported during monitoring. If these personnel possess the necessary competence, they can also assist with tasks such as cervical spine stabilization, suction, administering infusions, and more.

Question instructions: *What is your stance on the statement above?*

- ◯ Totally agree
- ◯ Partially agree
- ◯ Totally disagree

34 What do you want to change?

Thank you for your participation!
